# Supplementary material for: The relationship between psychological capital, patient’s contempt, and professional identity among general practitioners during COVID-19 in Chongqing, China
Source: PLoS One. 2023 Oct 9;18(10):e0287462. doi: 10.1371/journal.pone.0287462 (PMC10561861; doi:10.1371/journal.pone.0287462)
Supplement: S1 File — (DOCX) [file pone.0287462.s001.docx]

**S1 File 1: Identity professional scale**

Guidance: Please answer according to your own true feelings

| Scale | 1-strongly disagree, 2-Somewhat disagree, 3-neutral, 4-Somewhat agree, 5-strongly agree |
| --- | --- |
| 1. When referring to my profession, I usually say “we” rather than “they”. 2. I consider my success as the success of health care workers. 3. I care very much about other people’s views on my career. 4. The praise my profession from the people, which = is the praise they give on me. 5. If there are some criticisms on my career from the media, I will feel ashamed and embarrassed. 6. My job is important. /I believe in the importance of my job. 7. I am confident in my ability on work. 8. My job will exercise effects on patients’ conditions. 9. My job is meaningful. 10. I have the necessary qualifications and skills for my job. 11. I understand the responsibilities and requirements of the work. 12. Healthcare work suits me. 13. I know my role. | |

**Psychological capital scale**

Guidance: Please answer according to your own true feelings

| Scale | 1, strongly disagree; 2, disagree; 3, somewhat disagree; 4, somewhat agree; 5, agree; 6, strongly agree |
| --- | --- |
| 1.I believe I can analyze long-term problems and find solutions.  2.In meetings (with management, for example), I feel confident in stating what is within my scope of work.  3.I believe I can contribute to the discussion of the strategy of the company/community/organization.  4.In my line of work, I believe I can help set goals/goals.  5.I believe I can contact people outside (company/community/organization) (e.g. suppliers, customers, merchants) and discuss problems.  6.I am confident that I can present a message to a group of colleagues/officers.  7.If I find myself in a difficult situation at work, I can think of many ways to get out.  8.At present, I am full of energy to complete my work goals.  9.There are many solutions to any problem.  10.Right now, I think I'm pretty successful at my job.  11.I can think of many ways to achieve my current work goals.  12.At present, I am achieving the work goals I set for myself.  13.When I have a setback at work, it is difficult for me to recover from it and move on.  14.At work, I try to solve problems no matter what.  15.At work, if I have to do it, I can do it on my own, so to speak.  16.I usually take stress at work in stride.  17.Having been through a lot of tough times before, I am able to get through the tough times at work.  18.In my current job, I feel like I can juggle many things at once.  19.At work, when faced with uncertainty, I usually hope for the best.  20.If something can go wrong, even if I work wisely, it will go wrong.  21.I always look on the bright side of my work.  22.I am optimistic about what will happen to my job in the future.  23.In my current job, things have never worked out the way I wanted them to.  24.At work, I always believe that "behind the darkness is light, don't be pessimistic". | |

**Patient contempt scale**

Guidance: Please answer according to your own true feelings

| Scale | 1-strongly disagree, 2-Somewhat disagree, 3-neutral, 4-Somewhat agree, 5-strongly agree |
| --- | --- |
| 1.Some patients and their families distrust the rationality of their doctors' procedures.  2.Some patients and their families do not trust their doctors' skills and expertise.  3.Some patients and their families do not trust doctors' professional ethics.  4.Patients and their families often publicize negative opinions such as doctors receiving kickbacks, red envelopes, and lack of ethics.  5.Patients and their family members often behave disrespectfully to doctors during treatment.  6.The patients and their families think that doctors provide services because of their interests, which undermines the professional value of doctors.  7.Patients and their families lack the ability of empathy, ignoring the fact that doctors also need to survive and should respect.  8.In the process of doctor-patient disputes, patients and their families will choose to destroy and slander the social reputation of doctors.  9.In the minds of patients and their families, the professional image of doctors is not as good as before.  10.In the course of work, some professional suggestions of doctors are often questioned or even denied by patients and their families. | |
