# Supplementary material for: The relationship between psychological capital, patient’s contempt, and professional identity among general practitioners during COVID-19 in Chongqing, China
Source: PLoS One. 2023 Oct 9;18(10):e0287462. doi: 10.1371/journal.pone.0287462 (PMC10561861; doi:10.1371/journal.pone.0287462)
Supplement: Attachment — Submitted filename: Plos_ONE-D-22-31263_reviewer.pdf [file pone.0287462.s003.pdf]

PLOS ONE

The relationship between psychological capital,patient's contempt and professional identity among general practitioners in the era of COVID-19 in Chongqing,China

--Manuscript Draft--

**Manuscript Number:**	PONE-D-22-31263
**Article Type:**	Research Article
**Full Title:**	The relationship between psychological capital,patient's contempt and professional identity among general practitioners in the era of COVID-19 in Chongqing,China
**Short Title:**	The relationship between psychological capital, patient's contempt and professional identity
**Corresponding Author:**	Huisheng Deng<br>Chongqing Medical University First Affiliated Hospital<br>Chongqing, CHINA
**Keywords:**	general practice; professional identity; psychological capital; patient's contempt
**Abstract:**	General practitioners play a crucial role in the primary healthcare system and in epidemic prevention and control. However, few researchers have examined the professional identity of general practitioners. This study aims to investigate the current status of professional identity of general practitioners in Chongqing, China, and to explore the effects of psychological capital and patient’s contempt on their professional identity. A cross-sectional, online, self-assessed, questionnaire survey was conducted from December 2021 to January 2022. In total, 2,180 general practitioners working for more than one year were selected. Data were collected on participants’ sociodemographic characteristics, professional identity, psychological capital, and patient’s contempt. Multiple linear regression model was used to analyze the association between professional identity, psychological capital, patient’s contempt. The average score for professional identity among general practitioners was 53.59±6.42. The scores for self-efficacy, hope, optimism, and resilience (subscales of psychological capital) were 26.87±5.70, 26.47±5.74, 26.97±5.55, and 26.86±5.59, respectively. The score for perceived contempt was 34.19±7.59. An average monthly income greater than CNY8,000 ($\beta = 1.018$, $p < 0.001$), a work tenure higher than 15 years ($\beta = 0.440$, $p = 0.001$), hope ($\beta = 0.249$, $p < 0.001$), and a higher resilience score ($\beta = 0.333$, $p < 0.001$) were positively correlated with professional identity. Having a bachelor’s degree and above ($\beta = -0.720$, $p = 0.014$), an administrative role ($\beta = -1.456$, $p < 0.001$), self-efficacy ($\beta = -0.122$, $p < 0.001$), and higher patient’s contempt ($\beta = -0.103$, $p < 0.001$) were negatively associated with professional identity. General practitioners in Chongqing demonstrated high professional identity and a strong psychological status in the era of COVID-19. Psychological capital and patient’s contempt were associated with professional identity. To improve general practitioners’ professional identity, stakeholders should endeavor to promote practitioners’ mental health and physician–patient relationships in China.
**Order of Authors:**	Jingzhi Deng<br>Yang Xu<br>Qiaoya Li<br>Wen Yang<br>Huisheng Deng
**Additional Information:**	
**Question**	**Response**
**Financial Disclosure**<br><br>Enter a financial disclosure statement that describes the sources of funding for the work included in this submission. Review	HSD<br>2020ZLXM003<br>Joint project of Chongqing Health Commission and Science and Technology Bureau<br>http://wsjkw.cq.gov.cn/<br>http://kjj.cq.gov.cn/

Powered by Editorial Manager® and ProduXion Manager® from Aries Systems Corporation

the submission guidelines for detailed requirements. View published research articles from *PLOS ONE* for specific examples.<br><br>This statement is required for submission and **will appear in the published article** if the submission is accepted. Please make sure it is accurate.<br><br>**Unfunded studies**<br>Enter: *The author(s) received no specific funding for this work.*<br><br>**Funded studies**<br>Enter a statement with the following details:<br>• Initials of the authors who received each award<br>• Grant numbers awarded to each author<br>• The full name of each funder<br>• URL of each funder website<br>• Did the sponsors or funders play any role in the study design, data collection and analysis, decision to publish, or preparation of the manuscript?<br>• **NO** - Include this sentence at the end of your statement: *The funders had no role in study design, data collection and analysis, decision to publish, or preparation of the manuscript.*<br>• **YES** - Specify the role(s) played.<br><br>* typeset	The funders had a role in data collection of the manuscript.
**Competing Interests**<br><br>Use the instructions below to enter a competing interest statement for this submission. On behalf of all authors, disclose any competing interests that could be perceived to bias this work—acknowledging all financial support and any other relevant financial or non-financial competing interests.<br><br>This statement is required for submission and **will appear in the published article** if the submission is accepted. Please make sure it is accurate and that any funding sources listed in your Funding Information later in the submission form are also	The authors have declared that no competing interests exist.

declared in your Financial Disclosure statement.<br><br>View published research articles from *PLOS ONE* for specific examples.<br><br>**NO authors have competing interests**<br><br>Enter: *The authors have declared that no competing interests exist.*<br><br>**Authors with competing interests**<br><br>Enter competing interest details beginning with this statement:<br><br>*I have read the journal's policy and the authors of this manuscript have the following competing interests: [insert competing interests here]*<br><br>* typeset	
**Ethics Statement**<br><br>Enter an ethics statement for this submission. This statement is required if the study involved:<br><br>• Human participants<br>• Human specimens or tissue<br>• Vertebrate animals or cephalopods<br>• Vertebrate embryos or tissues<br>• Field research<br><br>Write "N/A" if the submission does not require an ethics statement.<br><br>General guidance is provided below. Consult the submission guidelines for detailed instructions. **Make sure that all information entered here is included in the Methods section of the manuscript.**	This study was approved by the institutional ethical review board of the first affiliated hospital of Chongqing medical university. During the investigation, doctors click the link to enter the homepage and see the informed consent form. If they click agree, the questionnaire content will be displayed for investigation; if they click disagree, the investigation will be finished. Electronic informed consent was obtained from all participants..

Powered by Editorial Manager® and ProduXion Manager® from Aries Systems Corporation

**Format for specific study types**<br><br>**Human Subject Research (involving human participants and/or tissue)**<br>• Give the name of the institutional review board or ethics committee that approved the study<br>• Include the approval number and/or a statement indicating approval of this research<br>• Indicate the form of consent obtained (written/oral) or the reason that consent was not obtained (e.g. the data were analyzed anonymously)<br><br>**Animal Research (involving vertebrate animals, embryos or tissues)**<br>• Provide the name of the Institutional Animal Care and Use Committee (IACUC) or other relevant ethics board that reviewed the study protocol, and indicate whether they approved this research or granted a formal waiver of ethical approval<br>• Include an approval number if one was obtained<br>• If the study involved *non-human primates*, add *additional details* about animal welfare and steps taken to ameliorate suffering<br>• If anesthesia, euthanasia, or any kind of animal sacrifice is part of the study, include briefly which substances and/or methods were applied<br><br>**Field Research**<br><br>Include the following details if this study involves the collection of plant, animal, or other materials from a natural setting:<br>• Field permit number<br>• Name of the institution or relevant body that granted permission	
**Data Availability**<br><br>Authors are required to make all data underlying the findings described fully available, without restriction, and from the time of publication. PLOS allows rare exceptions to address legal and ethical concerns. See the PLOS Data Policy and FAQ for detailed information.	Yes - all data are fully available without restriction

A Data Availability Statement describing where the data can be found is required at submission. Your answers to this question constitute the Data Availability Statement and **will be published in the article**, if accepted.<br><br>**Important:** Stating 'data available on request from the author' is not sufficient. If your data are only available upon request, select 'No' for the first question and explain your exceptional situation in the text box.<br><br>Do the authors confirm that all data underlying the findings described in their manuscript are fully available without restriction?	
**Describe where the data may be found in full sentences. If you are copying our sample text, replace any instances of XXX with the appropriate details.**<br><br>• If the data are **held or will be held in a public repository**, include URLs, accession numbers or DOIs. If this information will only be available after acceptance, indicate this by ticking the box below. For example: *All XXX files are available from the XXX database (accession number(s) XXX, XXX.).*<br>• If the data are all contained **within the manuscript and/or Supporting Information files**, enter the following: *All relevant data are within the manuscript and its Supporting Information files.*<br>• If neither of these applies but you are able to provide **details of access elsewhere**, with or without limitations, please do so. For example:<br><br>*Data cannot be shared publicly because of [XXX]. Data are available from the XXX Institutional Data Access / Ethics Committee (contact via XXX) for researchers who meet the criteria for access to confidential data.*<br><br>*The data underlying the results presented in the study are available from (include the name of the third party*	The datasets for this study can be made available by contacting the corresponding author.

and contact information or URL).<br>• This text is appropriate if the data are owned by a third party and authors do not have permission to share the data.<br><br>* typeset	
Additional data availability information:	

Manuscript Click here to access/download;Manuscript;manuscript.docx

The relationship between psychological capital, patient's contempt and professional identity among general practitioners in the era of COVID-19 in Chongqing, China

Jingzhi Deng[1¶], Yang Xu[1¶] , Qiaoya Li[2], Wen Yang[1], Huisheng Deng[1*]

[1] Department of General Practice, The First Affiliated Hospital of Chongqing Medical University, No. 1, Youyi Road, Chongqing 400016, People's Republic of China

[2] Department of General Practice, Yan'an Affiliated Hospital of Kunming Medical University, Kunming, 650051, People's Republic of China

*Corresponding author

E-mail:dhs700214@163.com(HD)

[¶]These authors contributed equally to this work.

Abstract

General practitioners play a crucial role in the primary healthcare system and in epidemic

prevention and control. However, few researchers have examined the professional identity of general practitioners. This study aims to investigate the current status of professional identity of general practitioners in Chongqing, China, and to explore the effects of psychological capital and patient's contempt on their professional identity. A cross-sectional, online, self-assessed, questionnaire survey was conducted from December 2021 to January 2022. In total, 2,180 general practitioners working for more than one year were selected. Data were collected on participants' sociodemographic characteristics, professional identity, psychological capital, and patient's contempt. Multiple linear regression model was used to analyze the association between professional identity, psychological capital, patient's contempt. The average score for professional identity among general practitioners was 53.59 ± 6.42. The scores for self-efficacy, hope, optimism, and resilience (subscales of psychological capital) were 26.87 ± 5.70, 26.47 ± 5.74, 26.97 ± 5.55, and 26.86 ± 5.59, respectively. The score for perceived contempt was 34.19 ± 7.59. An average monthly income greater than CNY8,000 ($\beta = 1.018$, $p < 0.001$), a work tenure higher than 15 years ($\beta = 0.440$, $p = 0.001$), hope ($\beta = 0.249$, $p < 0.001$), and a higher resilience score ($\beta = 0.333$, $p < 0.001$) were positively correlated with professional identity. Having a bachelor's degree and above ($\beta = -0.720$, $p = 0.014$), an administrative role ($\beta = -1.456$, $p < 0.001$), self-efficacy ($\beta = -0.122$, $p < 0.001$), and higher patient's contempt ($\beta = -0.103$, $p < 0.001$) were negatively associated with professional identity. General practitioners in Chongqing demonstrated high professional identity and a strong psychological status in the era of COVID-19. Psychological capital and patient's contempt were associated with professional identity. To improve general practitioners' professional identity, stakeholders should endeavor to promote practitioners'

mental health and physician–patient relationships in China.

Keywords: general practice, professional identity, psychological capital, patient’s contempt

Introduction

General practitioners (GPs), also known as family physicians, are the main providers of primary healthcare services and the “gatekeepers” of human health[1, 2]. Since the outbreak of COVID-19 in Wuhan, China, at the end of 2019, GPs have actively responded to the call of the country and played an indispensable role in prevention and control activities, such as the delivery of home care for community residents, screening of fever patients, and preventive triage[3-5]. Nonetheless, many countries, such as the United States, Australia, and South Korea, have long faced a shortage of GPs[6-8]. As the largest developing country in the world, the development of a general practice system in China came relatively late, and since the demand for primary healthcare services by the Chinese population has been increasing, the number of GPs is becoming relatively insufficient[9, 10].

The social identity of GPs tends to also be lower than that of specialist physicians. Because of their low social status, GPs are often easily ignored and not regarded as important in society, which also leads to a higher turnover rate among such professionals[1, 11]. GPs in China have also been in a high-intensity work environment for a long time now, making them generally currently suffer from both physical and psychological fatigue[12, 13]. If allowed to endure, these situations are likely to lead to a decline in GPs’ perception of and emotional

attachment to own profession, thus affecting their professional identity.

Professional identity refers to one's view of the goal, social value, and other related factors of an occupation, which tends to be consistent with the social evaluation and expectation of that occupation [14, 15]. This concept tends to indirectly reflect on students' transition to becoming physicians and dealing with the related daily life struggles [16]. Researchers show that professional identity impacts GPs' work engagement and career progress by affecting their sense of professional mission[17]. Higher professional identity can improve work quality and is conducive to patients' recovery from disease. However, there are relatively few investigations on the professional identity of GPs in China. According to a survey on the professional identity of GPs in China before the COVID-19 pandemic, the professional identity score of 3,236 GPs averaged at 51.23 (total score of 65); further, because of the regional economic development imbalance in China, the professional identity of GPs in Central China was significantly higher than that of GPs in other regions, and the professional identity of GPs with high income (vs. low income) and long working years (vs. low working years) was higher. Research also shows that foreign GPs identify more with their profession, and this may be because of the longer development time of the general practice system and the higher social status of GPs in foreign countries[18].

Psychological capital is a factor that has been shown to positively influence professional identity, being defined as positive psychological states related to the process of individual development[19-21]; it encompasses the concepts of self-efficacy, optimism, resilience, and hope. Self-efficacy refers to one's confidence in own competency for completing a task, facing challenges, and striving for success. Optimism refers to when a

person has a positive attribution style and attitude towards both the present and the future. Resilience is defined as the ability to quickly recover from adversity, setbacks, and failures, and even positively transform and grow after such happenings. Hope is a state of positive motivation to achieve a predetermined goal through various means[22].

GPs play the important role of ensuring citizen health management, meaning that they often need to deal with patients. However, due to knowledge inequality between physicians and patients, GPs may often not be understood nor respected by patients, and even suffer workplace violence. In fact, a mental health survey conducted with a sample of primary care physicians in China showed that 31.7% were facing mental health problems and depression[23]. Meanwhile, scholars have shown that positive psychological capital among GPs can influence negative emotional experiences in the work environment and help these professionals to deal with their negative emotions quicker[24, 25].

The emotional experiences of GPs with their patients during the COVID-19 pandemic also warrant attention. Harmonious physician–patient relationship is one of the foundational factors of GPs' professional identity[26]. However, contradictions between GPs and patients have long been a problem in the Chinese society[27, 28]. On the one hand, due to the imperfect Chinese primary healthcare system, patients in the country often face difficulties in seeing a medical practitioner. On the other hand, with the development of the Internet, patients sometimes prefer to seek help from the Internet instead of trusting medical practitioners[29]. Meanwhile, the latter are often worried about patients showing aggressive and threatening attitudes that endanger their safety, leading to the continuous deterioration of the physician–patient relationship.

For the purposes of this study, the aforementioned behaviors from patients that endanger the GPs' safety during physician–patient interactions will be referred to as "patient's contempt;" examples are GPs being disrespected or distrusted, and patients and their families showing non-cooperation and contempt during physician–patient interactions. We believe that patients' contempt can indirectly reflect the contradiction between physicians and patients and may affect the professional identity of GPs. Although the physician–patient relationship has improved in China since the outbreak of COVID-19, it remains an urgent problem that remains to be solved countrywide.

In summary, professional identity is extremely important to the quality of GPs' work, and despite this importance, few researchers have delved into the professional identity of these healthcare professionals. This study aimed to investigate the current status and relationship between psychological capital, patient's contempt and professional identity among GPs in Chongqing, China. It also attempted to explore the influencing factors of professional identity.

Methods

Study population and study design

A cross-sectional study on primary health care service centers in Chongqing, western

China, was conducted from December 2021 to January 2022 using stratified random sampling. Regarding the operationalization of the sampling of this study, first, information about the research was forwarded to the GP working group of Chongqing, China, through the staff at the administrative department of the Health Commission at the municipality. Second, the person in charge of each district or county forwarded the information to the person in charge of each primary health care service health center in the related area. Third, the person in charge of the health center selected the GPs who had worked there for more than one year. This process yielded a sample of 2,200 GPs from 23 districts. After excluding the data of 20 GPs with missing data or logic errors, a final sample of 2,180 valid questionnaires was obtained.

Ethics approval

This study was approved by the institutional ethical review board of the first affiliated hospital of Chongqing medical university. During the investigation, doctors click the link to enter the homepage and see the informed consent form. If they click agree, the questionnaire content will be displayed for investigation; if they click disagree, the investigation will be finished. Electronic informed consent was obtained from all participants.

Measures

Demographic characteristics

Regarding items on general demographic characteristics in the questionnaire, they included age, gender, educational background, marital status, contract status (GP wage,

welfare paid by the Chinese government, whether the health institution could freely fire GPs), work tenure, income level, professional title, having an administrative role, monthly frequency of working overtime, working hours, workplace, and frequency of family visits and of data collection.

Professional identity

For professional identity, we used the 13-item Chinese version of the professional identity scale from Zhao et al.'s and Wu's studies[30, 31] . It is rated on a 5-point Likert scale ranging from 1–5 (strongly disagree–strongly agree), and item scores are summed to provide a composite score of professional identity (range: 13–65), with higher scores representing higher professional identity. In the present study, the Cronbach's α for this scale was 0.91.

Psychological capital

Psychological capital was measured via the Psychological Capital Questionnaire (PCQ-24), which was developed by Luthans et al. and showed high internal consistency in their study [32]. It includes four dimensions, each with six items: self-efficacy, hope, resilience, and optimism. Items are rated on a scale ranging from 1–6 (1, strongly disagree; 2, disagree; 3, somewhat disagree; 4, somewhat agree; 5, agree; 6, strongly agree), and the higher the score, the stronger the psychological capital. In this study, the total Cronbach's α of the PCQ-24 scale was 0.933.

Patient's contempt

Zhang used key person interviews, focus group discussions, and a literature review to sort out, summarize, and determine the 10 common types of patient's contempt experienced

by medical practitioners[33]. Next, they designed a questionnaire of physicians' perceptions of patient's contempt, which showed a Cronbach's α of 0.935 in the original study. The questionnaire was scored on a 5-point scale (1, strongly disagree; 5, strongly agree), and total scores ranged from 10–50. The higher the score, the more serious the perception of patient's contempt by the medical practitioner.

Data collection and quality control

This study designed the questionnaire based on a literature review, group discussions, and simulation interviews. In order to improve the quality of the questionnaire, GPs in Chongqing Medical University conducted a preliminary experiment using it. Before the survey, they communicated with the leaders of the department. After obtaining consent, the investigators issued a Questionnaire Star QR code to the GPs in the department (a web link of the online questionnaire, created by the Questionnaire Star software, was then disseminated to GPs through WeChat, which is the biggest communication platform in China with over one billion users). The participants provided demographic information and their views on professional identity, psychological capital, and patient's contempt.

Statistical analysis

SPSS version 22.0 was used for data analysis. Qualitative data were described by the number of cases as a percentage (%),Quantitative data were described by the mean ± standard deviation ($x \pm s$). Independent-samples t-test, one-way analysis of variance was used to

compare the professional identity scores of different demographic characteristics. Multiple linear regression was used to compare the psychological capital , patient's contempt of different demographic characteristics. Pearson correlation analysis and multiple linear regression model was conducted to analyze the association between psychological capital , patient's contempt and professional identity. All tests were two-tailed, with a significance level of $p < 0.05$.

RESULTS

Sample and professional identity scores

Of 2,180 GPs, more than half were women (53.72%) and most were aged 30–49 years (67.1%). Furthermore, 64.95% of participants held a bachelor' s degree, 80.92% were married, 86.65% had contracts at the preparation stage, and 94.55% had an average monthly income of CNY≤8000. In addition, 53.07% held primary titles, 34.08% held intermediate titles, and 74.72% did not hold an administrative title. Finally, 97.57% of participants engaged in overtime work monthly, 682 (31.28%) had 15 years of tenure, and 71.97% of GPs in township hospitals undertook family visits and data collection. The scores of professional identity demonstrated statistically significant differences across age, gender, educational background, marital status, income level, professional title, management responsibility, and work tenure groups (all $p < 0.05$) (Table 1).

Table 1. The Professional Identity Scores of Different Demographic Characteristics

**Variables**	**N (%)**	**Score of professional identity**	**F/t**	**P**
**Age group (years)**			26.672	0.001

**＜30**	465 (21.30)	51.63±6.78		
**30~**	735 (33.70)	53.28±6.33		
**40~**	728 (33.40)	54.66±6.07		
**>50**	252 (11.60)	54.99±6.02		
**Sex**			2.917	0.004
**Men**	1009 (46.28)	54.02±6.47		
**Woman**	1171 (53.72)	53.22±6.36		
**Education level**			3.601	0.001
**Associate's degree or vocational diploma**	740 (33.94)	54.28±6.11		
**Bachelor degree or higher**	1416 (66.06)	53.24±6.55		
**Marital status**			5.263	0.001
**Married**	1764 (80.92)	53.94±6.27		
**Unmarried/widow/divorced**	416 (19.08)	52.11±6.82		
**Contract status**			-0.819	0.413
**Permanent**	1889 (86.65)	53.55±6.46		
**Temporary**	291 (13.35)	53.88±6.19		
**Average monthly income (CNY)**			29.37	0.001
**＜5000**	1096 (50.28)	52.64±6.51		
**5000~8000**	965 (44.27)	54.35±6.16		
**>8000**	119 (5.46)	56.20±6.20		
**Professional title**			8.215	0.001

**Elementary or below**	1157 (53.07)	53.19±6.54		
**Intermediate**	743 (34.08)	53.72±6.24		
**Senior**	280 (12.84)	54.89±6.22		
**Management responsibility**			6.716	0.001
**Yes**	551 (25.28)	55.16±6.23		
**No**	1629 (74.72)	53.06±6.40		
**Working overtime**			0.531	0.588
**Never**	53 (2.43)	54.25±6.70		
**Occasion**	943 (43.26)	53.68±5.99		
**Frequent**	1184 (54.31)	53.49±6.73		
**Work tenure (years)**			20.374	0.001
**＜5**	580 (26.61)	52.51±6.85		
**5~**	537 (24.63)	52.85±6.32		
**10~**	381 (17.48)	53.62±6.20		
**15~**	682 (31.28)	55.07±5.96		
**Practice setting**			-0.540	0.589
**Community health center**	829 (38.03)	53.50±6.49		
**Township health centers**	1351 (61.97)	53.65±6.38		
**Too many home visits and too much data sorting work**			0.641	0.521
**Yes**	1565 (71.79)	53.65±6.50		
**No**	615 (28.21)	53.45±6.21		

Analysis of the relationship between psychological capital and demographic characteristics

The scores for self-efficacy, hope, optimism, and resilience in psychological capital were 26.87±5.70, 26.47±5.74, 26.97±5.55, and 26.86±5.59, respectively. Table 2 shows the results of a multiple linear regression analysis to determine the related factors affecting the psychological capital of GPs. The psychological Capital of GPs was set as the dependent variable, while the other factors were set as independent variables. Age (β = 0.214, p = 0.003), marital status (β = -3.084, p = 0.012), income level (β = 3.108, p = 0.000), and overtime work (β = -3.399, p = 0.000) were the influencing factors of the GP psychological capital score ($p < 0.05$).

Table 2 Multiple linear regression between psychological capital and demographic characteristics

Variables	β	SE	T	P
**Age (years)**	0.214	0.072	2.969	0.003
**Sex**	-1.427	0.942	-1.516	0.130
**Education level**	-0.419	1.049	-0.399	0.690
**Marital status**	-3.084	1.220	-2.528	0.012
**Contract status**	2.646	1.357	1.950	0.051
**Average monthly income**	3.108	0.809	3.841	0.000
**Professional title**	-0.373	0.774	-0.483	0.629
**Management responsibility**	-0.409	1.102	-0.371	0.710
**Overtime work**	-3.399	0.828	-4.104	0.000

**Work tenure (years)**	0.916	0.481	1.904	0.057
**Practice setting**	1.829	0.956	1.913	0.056
**Too many home visits and too much data sorting work**	0.339	1.011	0.335	0.737

*Age＜30, male, associate's degree or vocational diploma, married, permanent, average monthly income＜5000, elementary or below, management responsibility (yes), never, work tenure＜5, community health center, and too many home visits and too much data sorting work (yes) were used as the control group.

Analysis of the relationship between patient's contempt and demographic characteristics

The score for patient's contempt was 34.19±7.59. Table 3 shows the results of a multiple linear regression analysis to determine the related factors affecting the patient's contempt of GPs. The patient's contempt of GPs was set as the dependent variable, while the other factors were set as independent variables. Age (β = -0.115, p = 0.000), sex (β = -1.570, p = 0.000), professional title (β =1.075, p = 0.000), management responsibility (β = 1.102, p = 0.000), and overtime work (β = 1.212, p = 0.000) were the influencing factors of the GP patient's contempt score ($p < 0.05$).

Table 3. Multiple linear regression between patient's contempt and demographic

characteristics.

Variables	β	SE	T	P
**Age (years)**	-0.115	0.027	-4.294	0.000
**Sex**	-1.570	0.351	-4.468	0.000
**Education level**	0.154	0.391	0.394	0.693
**Marital status**	-0.152	0.455	-0.335	0.738
**Contract status**	-0.361	0.506	-0.712	0.476
**Average monthly income**	-0.226	0.302	-0.749	0.454
**Professional title**	1.075	0.289	3.723	0.000
**Management responsibility**	1.102	0.411	2.680	0.007
**Overtime work**	1.212	0.309	3.919	0.000
**Work tenure (years)**	0.025	0.179	0.137	0.891
**Practice setting**	0.178	0.357	0.499	0.618
**Too many home visits and too much data sorting work**	-0.417	0.377	-1.105	0.269

*Age＜30, male, associate’s degree or vocational diploma, married, permanent, average monthly income＜5000, elementary or below, management responsibility (yes), never, work tenure＜5, community health center, and too many home visits and too much data sorting work (yes) were used as the control group.

Correlation analysis of psychological capital, perceived patient's contempt, and professional identity

Table 4 shows the correlation coefficients between professional identity, psychological capital,

and perceived patient's contempt. Specifically, the professional identity of GPs is positively correlated with the four dimensions of psychological capital and negatively correlated with patient's contempt (all $p < 0.01$).

Table 4. Correlation Analysis of Psychological Capital and Professional Identity.

	Self-efficacy	Hope	Optimism	Resilience	patient's contempt
**Professional identity**	0.257**	0.405**	0.393**	0.430**	-0.138**

* p<0.05 **p<0.01

Multiple linear regression for professional identity of GPs

Table 5 shows the results of a multiple linear regression analysis to determine the related factors associated with the professional identity of GPs. The GP professional identity score was set as the dependent variable, while the other factors were set as independent variables. Education level (β = -0.720, p = 0.014), income level (β = 1.018, p = 0.000), management responsibility (β = -1.456, p = 0.000), work tenure (β = 0.440, P = 0.001), self-efficacy (β = -0.122, p = 0.000), hope (β = 0.249, p = 0.000), resilience (β = 0.333, p = 0.000), and patient's contempt (β = -0.103, p = 0.000) were the influencing factors of the GPs' professional identity score ($p < 0.05$).

Table 5. Multiple Linear Regression for GP Professional Identity.

Variables	β	SE	T	P
**Age (years)***	-0.013	0.020	-0.656	0.512
**Sex***	0.038	0.265	0.145	0.885
**Education level***	-0.720	0.293	-2.461	0.014
**Marital status***	-0.393	0.341	-1.152	0.249
**Contract status***	0.345	0.379	0.909	0.363
**Average monthly income***	1.018	0.227	4.493	0.000
**Professional title***	-0.054	0.217	-0.248	0.804
**Management responsibility***	-1.456	0.308	-4.728	0.000
**Overtime work***	0.138	0.233	0.591	0.555
**Work tenure (years)***	0.440	0.134	3.275	0.001
**Practice setting***	-0.004	0.267	-0.014	0.989
**Too many home visits and too much data sorting work***	0.045	0.282	0.158	0.874
**Self-efficacy**	-0.122	0.032	-3.809	0.000
**Hope**	0.249	0.049	5.115	0.000
**Optimism**	-0.003	0.051	-0.064	0.949
**Resilience**	0.333	0.045	7.437	0.000
**patient's contempt**	-0.103	0.016	-6.376	0.000

*Age＜30, male, associate's degree or vocational diploma, married, permanent, average monthly income＜5000, elementary or below, management responsibility (yes), never, work tenure＜5, community health center, and too many home visits and too much data sorting work (yes) were used as the control group.

Discussion

The study, conducted in the context of the normalization of COVID-19, was the first to investigate the status and factors of GPs' professional identity, psychological capital, and patient's contempt in Chongqing, China. The research shows that the professional identity of GPs in Chongqing is high, but the mental health of GPs and the current doctor–patient environment continue to be defined by urgent issues that need attention. The professional identity of general practitioners is related to psychological capital and patients' contempt.

Our results show that the professional identity of GPs was proportional to their income level; the latter result is consistent with the findings of prior studies. Most GPs in China serve at the baseline level of the healthcare system and tend to have heavier workloads and lower incomes than medical specialists, Previous studies showed that a high income can help GPs overcome life difficulties, ensure self-worth, and indirectly increase their professional identity[34-36].

GPs with more working years had stronger senses of professional identity than those with less working years, and the increase in work tenure had a significant role in promoting professional identity. A potential reason for this is that physicians have longer working hours than those in other occupations; this may cause them to experience more job-related pressures early on in their careers, which can lead to job burnout[37, 38]. This may also explain why the degree of professional identity in the early career stage was generally low. As work tenure increases, GPs gain a deeper understanding of their occupation, increase their performance

quality, and obtain a greater sense of achievement in their work, which may reduce job burnout and improve their enthusiasm for their work; in turn, these changes may enable them to feel a stronger sense of professional identity .

GPs who held administrative roles had a stronger sense of professional identity than those who did not. This may be because GPs in administrative positions have more opportunities for career development, which may contribute to their job satisfaction and sense of personal value[39]. At the same time, the higher the GP's educational background, the poorer their sense of professional identity; this may be because GPs with higher levels of education are usually assigned to administrative positions, which may cause them to feel as if they are not progressing in their careers. Additionally, the combination of higher education and the relatively large amount of responsibly that characterize GPs in administrative positions responsibilities, which can hinder their professional knowledge development. If these assumptions are accurate, this may be a problem that will require more work from interested parties at the grassroots level to solve. Based on these findings, managers can take corresponding measures and strategies to stabilize the professional identity of GPs; for example, they may do so by reasonably increasing GP income, regularly checking GP mental health, and creating a harmonious doctor–patient environment.

Meanwhile, this study also found that age, marital status, average monthly income, and overtime work were related to psychological capital. As they age, GPs gradually transition from students to practitioners. During this shift, they increase their connections with patients in their daily work, often become more flexible in dealing with different issues, and gradually become more mature.

Regarding marital status, being married was shown to strengthen psychological capital; this may be because married GPs may be able to enjoy timely support from their partners and families when they encounter unpleasant situations in life[22]. This may, in turn, reduce their incidence of depression and strengthen their psychological capital.

Moreover, an increase in income was also shown to promote psychological capital. In China, it is generally believed that GPs are not as prestigious as specialist physicians, leading to their low social status and income. Furthermore, because the growth cycle for this medical career is relatively long, GPs need to work and do research in order to accelerate their promotions[40]. Nevertheless, GPs that work in relatively large hospitals have access to fewer resources, which can make their road to promotion more difficult. When this is coupled with the efforts that early career GPs tend to make, fatigue can occur and their efforts can fall out of step with their return, This reality may also lead GPs with lower psychological capital to have lower income[41].

Additionally, GPs in our sample who frequently worked overtime were associated with lower levels of psychological capital[42]. Compared with medical specialists, who tend to focus on the treatment of specific diseases, GPs' daily work requires them to think about several different patient needs in a high-pressure work environment. Considering these aspects of their work, GPs may be likely to experience mood disorders or symptoms, such as depressive episodes or anxiety. If these are not tackled appropriately and timely, then GPs' psychological capital may decline.

Many previous studies have shown that higher psychological capital can play a positive role in how GPs treat their patients during a consultation, significantly improve

negative job-related emotions, and enable more proactive behaviors. Because GPs must engage a large number of patients every day, they inevitably encounter patients who may not like them or treat them kindly, GPs with stronger psychological structures may be able to get over such situations more quickly than others and may also be more likely to deal with such situations without experiencing depression or depressive symptoms[43].

In China, the current situation regarding patients' distrust of physicians has become so problematic that physician–patient conflicts often emerge; this, in turn, leads to an increase in patients' doubts about their GPs' professionalism and skills, which can negatively impact patient prognosis. Specifically, while GPs often try to solve their patients' problems, their patients may perceive their treatments as unsatisfactory，Leading GPS to doubts about own their own skills and abilities with poor professional identity[44]. Particularly, in bad long-term physician–patient relationships, GPs' positive feelings regarding their profession may diminish, decreasing their professional identity, increasing doubts in professional skills, and increasing their turnover rate. Our results are not entirely concordant with prior evidence, as self-efficacy negatively influenced professional identity among our sample of GPs[45, 46]. This may be related to the Chinese setting of the study; specifically, Chinese GPs may be confident in their practical abilities, but the physician–patient relationships they encounter may be characterized by a lack of trust. Patients often have doubts about doctors' treatment and feel that doctors do not try their best to solve problems for themselves, which leads to unsatisfactory curative effect in the treatment process. For a long time, doctors will have doubts about their professional ability and lead to the decline of their professional identity.

This study also shows that higher hope and resilience are associated with improved

GP professional identity. Thus, positive psychology may help GPs adjust to the pressures they experience during clinical practice, increase investment in work, and affect their performance, potentially enabling psychological well-being in daily work and promoting job re-engagement [47].

Furthermore, the survey results show that GPs with higher patient’s contempt scores had lower professional identity. Before the new health care reform in China was implemented, some widespread phenomena (e.g., doctors being given rebates by prescribing designated drugs to patients, patients giving doctors monetary gifts) resulted in increasingly tense doctor-patient relationships and progressively increased patient distrust [48, 49]. In foreign countries, GP are relatively older and more experienced, which can usually better help patients solve medical problems. However, in China, due to the late establishment of the GP system and its imperfections, most GPs are young people who have just graduated from medical colleges. Compared with older GPs, they have little clinical knowledge; this can cause them to have a poorer ability to carry out professional processes, which indirectly increases the unfriendly relationship between doctors and patients. The physician–patient relationship is a special interpersonal relationship, and good communication can improve it and enable patients to shift from passively to actively engaging in their treatment. Through patients’ active participation, GPs can better understand their actual needs, solve problems from the patient’s perspective, obtain patient understanding and trust, and gradually decrease patient’s contempt regarding medical care and practitioners [50, 51]. In such scenarios, the physician–patient relationship becomes more cooperative, and when then the goals and efforts of both GPs and patients become more consistent, the physician–patient relationship

may improve and physicians' perceptions of patient's contempt may diminish. Promoting cooperation in physician–patient relationships may also help GPs to more clearly recognize the value of their profession and improve their professional identity.

Limitations

However, this study had some limitations. First, this study adopted a cross-sectional design; therefore, it was not able to determine causal relationships between the variables of interest. Researchers should use prospective cohort study designs in the future to expand our conclusions. Second, other potential predictors of GP professional identity (e.g., work stress, workplace violence, team support) were not included in our questionnaire, and future researchers should further explore the influence of these factors on professional identity in our study. Third, our sample is from a municipality in western China, which means it may not be applicable to the situation of GPs across the country; in the future, the sample size should be expanded.

Conclusions

The normalization of the COVID-19 pandemic has brought great challenges to China'' primary health system, within which GPs are an indispensable force. GPs' sense of professional identity affects their daily work efficiency and stability. This study found that income level, position type (administrative/non-administrative), work duration, self-efficacy, hope, resilience, and contempt for patients affect professional identity among GPs in China.

If managers fail to pay attention to these factors in a timely manner, GP talent may be lost. These survey results can provide a basis for relevant departments to formulate specific strategies for GP. At the individual level, GPs should strengthen their psychology by developing a positive and optimistic attitude toward difficulties. Efforts should be made at the national level to build a friendly doctor–patient environment. Notably, these interventions can improve general practice and reduce the brain drain.

Acknowledgments

We want to thank Chongqing Health Commission and Science and Technology Bureau for support in data acquisition.

References

1. Gan Y, Gong Y, Chen Y, Cao S, Li L, Zhou Y, et al. Turnover intention and related factors among general practitioners in Hubei, China: a cross-sectional study. 2018;19(1):74. doi: 10.1186/s12875-018-0752-3. PubMed PMID: 29793443.
2. Sun Y, Luo Z, Fang P. Factors influencing the turnover intention of Chinese community health service workers based on the investigation results of five provinces. J Community Health. 2013;38(6):1058-66. doi: 10.1007/s10900-013-9714-9. PubMed PMID: 23775034.
3. Gruda D, Kafetsios KJIjoer, health p. I Need a Doctor, Call Me a Doctor: Attachment and the Evaluation of General Practitioners before and during the COVID-19 Pandemic. 2021;18(15). doi: 10.3390/ijerph18157914. PubMed PMID: 34360207.
4. Dujardin J, Schuurmans J, Westerduin D, Wichmann A, Engels YJPm. The COVID-19 pandemic: A tipping point for advance care planning? Experiences of general practitioners. 2021;35(7):1238-48. doi: 10.1177/02692163211016979. PubMed PMID: 34041987.

5. Liu Y, Wang Z, Ren J, Tian Y, Zhou M, Zhou T, et al. A COVID-19 Risk Assessment Decision Support System for General Practitioners: Design and Development Study. 2020;22(6):e19786. doi: 10.2196/19786. PubMed PMID: 32540845.

6. Sammut M, Lindh M, Rindlisbacher B, JTEjogp. Funding of vocational training programmes for general practice/family medicine in Europe. 2008;14(2):83-8. doi: 10.1080/13814780802095576. PubMed PMID: 18609349.

7. Kilo C, Wasson JJHa. Practice redesign and the patient-centered medical home: history, promises, and challenges. 2010;29(5):773-8. doi: 10.1377/hlthaff.2010.0012. PubMed PMID: 20439860.

8. Shin HJBfp. A survey of the working status of family medicine physicians in clinics and hospitals in Korea. 2020;21(1):82. doi: 10.1186/s12875-020-01154-5. PubMed PMID: 32384873.

9. Lian S, Chen Q, Yao M, Chi C, Fetters MJFm. Training Pathways to Working as a General Practitioner in China. 2019;51(3):262-70. doi: 10.22454/FamMed.2019.329090. PubMed PMID: 30861081.

10. Zhu J, Ariana PJBo. Provider perspectives on general practice in Henan, China: a mixed-methods study. 2020;10(2):e036240. doi: 10.1136/bmjopen-2019-036240. PubMed PMID: 32019820.

11. Kroneman M, Meeus P, Kringos D, Groot W, van der Zee JJBhsr. International developments in revenues and incomes of general practitioners from 2000 to 2010. 2013;13:436. doi: 10.1186/1472-6963-13-436. PubMed PMID: 24152337.

12. Lai J, Ma S, Wang Y, Cai Z, Hu J, Wei N, et al. Factors Associated With Mental Health Outcomes Among Health Care Workers Exposed to Coronavirus Disease 2019. 2020;3(3):e203976. doi: 10.1001/jamanetworkopen.2020.3976. PubMed PMID: 32202646.

13. Tian F, Shu Q, Cui Q, Wang L, Liu C, Wu HJFiph. The Mediating Role of Psychological Capital in the Relationship Between Occupational Stress and Fatigue: A Cross-Sectional Study Among 1,104 Chinese Physicians. 2020;8:12. doi: 10.3389/fpubh.2020.00012. PubMed PMID: 32185156.

14. Fitzgerald A. Professional identity: A concept analysis. Nursing Forum. 2020;55(3):447-72. doi: 10.1111/nuf.12450. PubMed PMID: 32249453.

15. Ohlén J, Segesten K. The professional identity of the nurse: concept analysis and development. J Adv Nurs. 1998;28(4):720-7. PubMed PMID: 9829659.

16. Deng S, Yang N, Li S, Wang W, Yan H, Li H. Doctors' Job Satisfaction and Its Relationships With Doctor-Patient Relationship and Work-Family Conflict in China: A Structural Equation Modeling. Inquiry. 2018;55:46958018790831. doi: 10.1177/0046958018790831. PubMed PMID: 30371128.

17. Scanlan JN, Hazelton T. Relationships between job satisfaction, burnout, professional identity and meaningfulness of work activities for occupational therapists working in mental health. Aust Occup Ther J. 2019;66(5):581-90. doi: 10.1111/1440-1630.12596. PubMed PMID: 31304598.

18. Li L, Gan Y, Yang Y, Jiang H, Lu K, Zhou X, et al. Analysis on professional identity and related factors among Chinese general practitioners: a National Cross-sectional Study. BMC Family Practice. 2020;21(1):80. doi: 10.1186/s12875-020-01155-4. PubMed PMID: 32375674.

19. Qiu T, Liu C, Huang H, Yang S, Gu Z, Tian F, et al. The mediating role of psychological capital on the association between workplace violence and professional identity among Chinese doctors: a cross-sectional study. 2019;12:209-17. doi: 10.2147/prbm.S198443. PubMed PMID: 31114405.

20. Ren Z, Zhang X, Li X, He M, Shi H, Zhao H, et al. Relationships of organisational justice, psychological capital and professional identity with job burnout among Chinese nurses: A cross-sectional study. 2021;30:2912-23. doi: 10.1111/jocn.15797. PubMed PMID: 33829587.

21. Hao C, Zhu L, Zhang S, Rong S, Zhang Y, Ye J, et al. Serial Multiple Mediation of Professional Identity, and Psychological Capital in the Relationship Between Work-Related Stress and Work-Related Well-Being of ICU

Nurses in China: A Cross-Sectional Questionnaire Survey. 2020;11:535634. doi: 10.3389/fpsyg.2020.535634. PubMed PMID: 33414737.

22. Xu N, Zhao S, Xue H, Fu W, Liu L, Zhang T, et al. Associations of perceived social support and positive psychological resources with fatigue symptom in patients with rheumatoid arthritis. PLoS One. 2017;12(3):e0173293. doi: 10.1371/journal.pone.0173293. PubMed PMID: 28291837.

23. Shen L-L, Lao L-M, Jiang S-F, Yang H, Ren L-M, Ying DG-C, et al. A survey of anxiety and depression symptoms among primary-care physicians in China. International Journal of Psychiatry In Medicine. 2012;44(3):257-70. PubMed PMID: 23586280.

24. Xu H, Liu X, Zeng PJPr, management b. The Mediating Role of Social Support in the Relationship Between Psychological Capital and Depression Among Chinese Emergency Physicians. 2022;15:977-90. doi: 10.2147/prbm.S360611. PubMed PMID: 35480711.

25. Jin J, Li H, Song W, Jiang N, Zhao W, Wen DJMeo. The mediating role of psychological capital on the relation between distress and empathy of medical residents: a cross-sectional survey. 2020;25(1):1710326. doi: 10.1080/10872981.2019.1710326. PubMed PMID: 31900104.

26. Muszbek K, Gaal I. [Pitfalls within the cancer-related doctor-patient communication]. Orv Hetil. 2016;157(17):649-53. doi: 10.1556/650.2016.30454. PubMed PMID: 27084437.

27. Qiao T, Fan Y, Geater AF, Chongsuvivatwong V, McNeil EB. Factors associated with the doctor-patient relationship: doctor and patient perspectives in hospital outpatient clinics of Inner Mongolia Autonomous Region, China. Patient Prefer Adherence. 2019;13:1125-43. doi: 10.2147/PPA.S189345. PubMed PMID: 31409976.

28. Xu B. The impact of COVID-19 on the doctor-patient relationship in China. Frontiers In Public Health. 2022;10:907009. doi: 10.3389/fpubh.2022.907009. PubMed PMID: 35979467.

29. Freckelton IR. Internet Disruptions in the Doctor-Patient Relationship. Med Law Rev. 2020;28(3):502-25. doi: 10.1093/medlaw/fwaa008. PubMed PMID: 32417891.

30. Zhao H, Lu T, Zhang C, Deng F, Zheng J. Testing for reliability and validity of Chinese version of the nurse's career identity scale. Chinese Nurs Manag. 2010;10(11):49–51.

31. Wu H. Public hospital medical workers’ prefessional identity research. Chengdu: Southwestern University of Finance and Economics; 2012.

32. Luthans F, Youssef C M, Avolio B J:Psychological capital: Developing the human competitive edge. Oxford, UK: Oxford University Press;2007.

33. Zhang S, Liu G, Zhang P, Yin H,Zhao X, Wang H, Ji K, Chen S, Zhang X: Impact of perceived belittlement from patient on the career identity and turnover intention of chinese doctors. Chinese Hospital Management. 2021;41(10):55-58.

34. Correia I, Almeida AE. Organizational Justice, Professional Identification, Empathy, and Meaningful Work During COVID-19 Pandemic: Are They Burnout Protectors in Physicians and Nurses? Frontiers In Psychology. 2020;11:566139. doi: 10.3389/fpsyg.2020.566139. PubMed PMID: 33362629.

35. Oh Y-I, Kim H, Kim K. Factors Affecting Korean Physician Job Satisfaction. International Journal of Environmental Research and Public Health. 2019;16(15). doi: 10.3390/ijerph16152714. PubMed PMID: 31366037.

36. Shibasaki I, Usui A, Morita S, Motomura N, Haruyama Y, Yokoyama H. Sociodemographic and work-related factors influencing long working hours among cardiovascular surgeons in Japan: a cross-sectional study. Ind Health. 2022;60(1):16-28. doi: 10.2486/indhealth.2021-0039. PubMed PMID: 34629368.

37. Mukherjee S, Meacock J, Kissane E, Pal D. Factors relating to working hours restriction that have impacted the professional identity of trainees in the last decade. Br J Hosp Med (Lond). 2021;82(3). doi:

10.12968/hmed.2020.0355. PubMed PMID: 33792379.
38. Carvajal MJ, Popovici I, Hardigan PC. Gender and Pharmacists' Career Satisfaction in the United States. Pharmacy (Basel, Switzerland). 2021;9(4). doi: 10.3390/pharmacy9040173. PubMed PMID: 34698292.
39. Yang S, Huang H, Qiu T, Tian F, Gu Z, Gao X, et al. Psychological Capital Mediates the Association Between Perceived Organizational Support and Work Engagement Among Chinese Doctors. Frontiers In Public Health. 2020;8:149. doi: 10.3389/fpubh.2020.00149. PubMed PMID: 32528918.
40. Emery SE, Gregory C. Physician incentives for academic productivity. An analysis of orthopaedic department compensation strategies. J Bone Joint Surg Am. 2006;88(9):2049-56. PubMed PMID: 16951123.
41. Yıldırım N, Coşkun H, Polat Ş. The Relationship Between Psychological Capital and the Occupational Psychologic Risks of Nurses: The Mediation Role of Compassion Satisfaction. J Nurs Scholarsh. 2021;53(1):115-25. doi: 10.1111/jnu.12607. PubMed PMID: 33146952.
42. Britt TW. Aspects of Identity Predict Engagement in Work Under Adverse Conditions. Self and Identity. 2003;2(1):31-45. doi: 10.1080/15298860309022.
43. Tei-Tominaga M, Nakanishi M. The Influence of Supportive and Ethical Work Environments on Work-Related Accidents, Injuries, and Serious Psychological Distress among Hospital Nurses. International Journal of Environmental Research and Public Health. 2018;15(2). doi: 10.3390/ijerph15020240. PubMed PMID: 29385044.
44. Qiu T, Liu C, Huang H, Yang S, Gu Z, Tian F, et al. The mediating role of psychological capital on the association between workplace violence and professional identity among Chinese doctors: a cross-sectional study. Psychol Res Behav Manag. 2019;12:209-17. doi: 10.2147/PRBM.S198443. PubMed PMID: 31114405.
45. Ren Z, Zhang X, Li X, He M, Shi H, Zhao H, et al. Relationships of organisational justice, psychological capital and professional identity with job burnout among Chinese nurses: A cross-sectional study. J Clin Nurs. 2021;30(19-20):2912-23. doi: 10.1111/jocn.15797. PubMed PMID: 33829587.
46. Hao C, Zhu L, Zhang S, Rong S, Zhang Y, Ye J, et al. Serial Multiple Mediation of Professional Identity, and Psychological Capital in the Relationship Between Work-Related Stress and Work-Related Well-Being of ICU Nurses in China: A Cross-Sectional Questionnaire Survey. Frontiers In Psychology. 2020;11:535634. doi: 10.3389/fpsyg.2020.535634. PubMed PMID: 33414737.
47. Xu X, Zhou L, Asante-Antwi H, Boafo-Arthur A, Mustafa T. Reconstructing family doctors' psychological well-being and motivation for effective performance in China: the intervening role of psychological capital. BMC Family Practice. 2020;21(1):137. doi: 10.1186/s12875-020-01182-1. PubMed PMID: 32650723.
48. Jing W, Otten H, Sullivan L, Lovell-Simons L, Granek-Catarivas M, Fritzsche K. Improving the doctor-patient relationship in China: the role of balint groups. International Journal of Psychiatry In Medicine. 2013;46(4):417-27. PubMed PMID: 24922991.
49. Li L, Fu H. China's health care system reform: Progress and prospects. Int J Health Plann Manage. 2017;32(3):240-53. doi: 10.1002/hpm.2424. PubMed PMID: 28612498.
50. Wang J, Zheng Q, Song W, Wei L. The Effect of Nursing Students' Self-Efficacy on Patient-Centered Communication During the COVID-19 Pandemic: The Mediating Effect of Learning Burnout. Frontiers In Psychiatry. 2021;12:787819. doi: 10.3389/fpsyt.2021.787819. PubMed PMID: 34925111.
51. Ghaffarifar S, Ghofranipour F, Ahmadi F, Khoshbaten M, Sallis C. The Causal Relationship between Interns' Knowledge and Self-Efficacy and Their Value in Predicting the Interns' Communication Behavior with Patients. Int J Community Based Nurs Midwifery. 2015;3(4):263-71. PubMed PMID: 26448953.

Click here to access/download
Supporting Information
S1 scale.docx
