# Supplementary material for: The relationship between psychological capital, patient’s contempt, and professional identity among general practitioners during COVID-19 in Chongqing, China
Source: PLoS One. 2023 Oct 9;18(10):e0287462. doi: 10.1371/journal.pone.0287462 (PMC10561861; doi:10.1371/journal.pone.0287462)
Supplement: Attachment — Submitted filename: Reviewer.docx [file pone.0287462.s004.docx]

I found this study quite interesting. Congratulations to the authors. I just have a very small suggestion.

Introduction

“Self-efficacy refers to one’s confidence in own competency for completing a task, facing challenges, and striving for success. Optimism refers to when a person has a positive attribution style and 90 attitude towards both the present and the future. Resilience is defined as the ability to quickly recover from adversity, setbacks, and failures, and even positively transform and grow after such happenings. Hope is a state of positive motivation to achieve a predetermined goal through various means[22].”

This paragraph breaks the integrity of the introduction. My suggestion: 94.-101. It can be added to the end of the paragraph in the page range.

Methods

Cronbach's alpha values of the scales should be given.
