# Supplementary material for: The relationship between psychological capital, patient’s contempt, and professional identity among general practitioners during COVID-19 in Chongqing, China
Source: PLoS One. 2023 Oct 9;18(10):e0287462. doi: 10.1371/journal.pone.0287462 (PMC10561861; doi:10.1371/journal.pone.0287462)
Supplement: Attachment — Submitted filename: Response to Reviewers.docx [file pone.0287462.s005.docx]

**Response to the Editor and Reviewers**

We thank both the editor and the reviewers for their positive and constructive comments and suggestions.

**Response to the Editor’s note and suggestions**

We have uploaded the minimal anonymized data as Supporting Information files. Thank you for your advice.

**Response to Reviewer 1**

Comment 1:A reference on the type of choice of population must be met here: convenience? random?

Response 1: Thank you for your advice. We have added to the sentence an explanation of how the population was chosen; the population was selected randomly.

Comment 2:The specific scales used must be mentioned in the abstract.

Response 2: Thank you for your constructive comments. We have addressed the problem you mentioned. The specific scales used are now stated in the abstract, which has been modified as follows: "General practitioners’ sense of professional identity, mental health, and sense of patients' disrespect were measured using the Professional Identity Scale, Psychological Capital Questionnaire, and Patient's Contempt Questionnaire. Sociodemographic characteristics were also collected.” (Page 2, lines 32-35)

Comment 3: Validity of all scales used, namely the Cronbach's alfa shoulde be refered.

Response 3: Thank you for your suggestion. We have calculated Cronbach's alpha of all scales in the Measures section and added them to the end of the respective sentences. The Cronbach's alpha values of the Professional Identity Scale, Psychological Capital Questionnaire, and Patient's Contempt Questionnaire were 0.936, 0.977, and 0.914, respectively.

Comment 4:responsibility... butthis sentende must be corrected.

Response 4: Thank you for your comments. We also see the problem with this sentence. Therefore, we have revised to "General practitioners with higher education are subject to higher social expectations; thus, they hope to obtain corresponding social status. " (Page 19, lines 310-313)

Comment 5:The second GPS must be altered to GPs. Could a mention about the patiente-doctor relationship be included? How come this is about not using Patient Centered Medicine? How come this is due to over use of medicines and under expeted use of analysis and othe neans of study like radiographs and other exmas? What about the media's press on patients?

Response 5: Thank you for your opinion, which is very helpful in the improvement of our article. We have revised the second GPS to GPs. Regarding your reference to the sentence "While GPs often try to solve their patients' problems, their patients may perceive their treatments as unsatisfactory, leading GPs to doubts about own their own skills and abilities with poor professional identity," you asked whether this sentence can be considered from other perspectives. After consideration, we think that doing so is highly necessary; thus, we read relevant references and added a supplementary explanation of this sentence, hoping to meet your requirements. (Pages 21-22, lines 357-377)

References

44.	de Waard CS, Poot AJ, den Elzen WPJ, Wind AW, Caljouw MAA, Gussekloo J. Perceived doctor-patient relationship and satisfaction with general practitioner care in older persons in residential homes. Scand J Prim Health Care. 2018;36(2):189-97. doi: 10.1080/02813432.2018.1459229. PubMed PMID: 29644911.

45.	Zhong C, Zhou M, Luo Z, Liang C, Li L, Kuang L. Association between doctor-patient familiarity and patient-centred care during general practitioner's consultations: a direct observational study in Chinese primary care practice. BMC Fam Pract. 2021;22(1):107. doi: 10.1186/s12875-021-01446-4. PubMed PMID: 34049489.

46.	Zhou Y, Ma Y, Yang WFZ, Wu Q, Wang Q, Wang D, et al. Doctor-patient relationship improved during COVID-19 pandemic, but weakness remains. BMC Fam Pract. 2021;22(1):255. doi: 10.1186/s12875-021-01600-y. PubMed PMID: 34937550.

47.	Zhou Y, Yang WFZ, Ma Y, Wu Q, Yang D, Liu T, et al. Doctor-Patient Relationship in the Eyes of Medical Professionals in China During the COVID-19 Pandemic: A Cross-Sectional Study. Front Psychiatry. 2021;12:768089. doi: 10.3389/fpsyt.2021.768089. PubMed PMID: 34777069.

48.	Zhou Y, Chen S, Liao Y, Wu Q, Ma Y, Wang D, et al. General Perception of Doctor-Patient Relationship From Patients During the COVID-19 Pandemic in China: A Cross-Sectional Study. Front Public Health. 2021;9:646486. doi: 10.3389/fpubh.2021.646486. PubMed PMID: 34295863.

Comment 6:I do not think you have a limitation here! What other ways do you have to undestand a problem? A prospective study would mean a first picture which is what you have done. We can only see a problem when we picture it. Then we can intervene.

Response 6: Thank you for your constructive comments. We have revised the limitations according to your suggestion.

**Response to Reviewer 2**

Comment 1:Introduction:“Self-efficacy refers to one’s confidence in own competency for completing a task, facing challenges, and striving for success. Optimism refers to when a person has a positive attribution style and 90 attitude towards both the present and the future. Resilience is defined as the ability to quickly recover from adversity, setbacks, and failures, and even positively transform and grow after such happenings. Hope is a state of positive motivation to achieve a predetermined goal through various means[22].”This paragraph breaks the integrity of the introduction. My suggestion: 94.-101. It can be added to the end of the paragraph in the page range.

Response 1: Thank you for your constructive comments. We noticed that the question you raised is very helpful in improving the quality of our article. Therefore, we have adjusted the word order of this portion of the sentence to address your concerns.

Comment 2:Methods:Cronbach’s alpha values of the scales should be given.

Response 2: Thank you for your advice. We have calculated the Cronbach's alpha of all scales in the Measures section and added them to the end of the respective sentences. The Cronbach's alpha values of the Professional Identity Scale, Psychological Capital Questionnaire, and Patient's Contempt Questionnaire were 0.936, 0.977, and 0.914, respectively.
