# Supplementary material for: The relationship between psychological capital, patient’s contempt, and professional identity among general practitioners during COVID-19 in Chongqing, China
Source: PLoS One. 2023 Oct 9;18(10):e0287462. doi: 10.1371/journal.pone.0287462 (PMC10561861; doi:10.1371/journal.pone.0287462)
Supplement: Attachment — Submitted filename: Response_to_Reviewers.docx [file pone.0287462.s006.docx]

**Response to the Editor and Reviewers**

We thank both the editor and the reviewers for their positive and constructive comments and suggestions.

**Response to the Editor’s note and suggestions**

Comment 1. There are no examples in your measurement tool.

Response 1: Thank you for your comment. We have added examples of the measurement tool (lines 160–185).

Comment 2. Lack of research hypotheses.

Response 2: Thank you for your comment. We hypothesized that the professional identity of general practitioners would be improved during COVID-19, and greater psychological capital would promote the professional identity of these persons, while patient’s contempt might lead to a reduction in the professional identity of general practitioners. We have added this sentence to the article (lines 125–128).

Comment 3. The author adopted a sampling method (stratified random sampling) with probability, but the expression in the paper does not show probability.

Response 3: Thank you for your comment. We apologize for our lack of clarity. We adopted a randomized cluster sampling method, in which we selected 23 districts and counties; we recruited all general practitioners who met the inclusion and exclusion criteria in the selected areas(lines 133-143).

Comment 4. Lack of convergence validity and discriminative validity tests.

Response 4: Thank you for noting these issues. The AVE, CR and sqrt(AVE) value of the Professional Identity Scale are 0.450,0.910,0.671. The Psychological Capital Questionnaire includes four dimensions: self-efficacy, hope, resilience and optimism. The AVE, CR, and sqrt(AVE) values are 0.596,0.897,0.772 in the self-efficacy dimension; 0.650,0.918,0.806 in the hope dimension; 0.682,0.927,0.826 in the resilience dimension; 0.711,0.937,0.843 in the optimism dimension.The AVE, CR and sqrt(AVE) value of the Patient’s Contempt Questionnaire are0.543,0.922,0.737.The convergence validity and discriminative validity of the questionnaire are reliable.

Comment 5. Please refer to other papers and revised the presentation of Table 4, which is inconsistent with the common correlation analysis.

Response 5: Thank you for your constructive comment. We have read other articles and modified Table 4 accordingly, which improved the presentation of our article.

Comment 6. Lack of CMV analysis.

Response 6: Thank you for your advice. We conducted CMV analysis of the questionnaire data via Harman's single-factor test. The exploratory factor analysis results for the 47 items revealed 7 factors with an eigenvalue greater than 1. The variance explained by the first factor was 34.148% (<50%), indicating no serious common method bias in this study.

Comment 7. Lines 295-299, "A potential reason for this is that physicians have longer working hours than those in other occupations; this may cause them to experience more job-related pressures early on in their careers, which can lead to job burnout" .This sentence is very farfetched in explaining the previous sentence.

Response 7: Thank you for your constructive comment. After reading the relevant literature, we changed this sentence to " The professional identity of GPs with more work experience is stronger than that of GPs with fewer years of experience; increased years working significantly promotes professional identity. When young doctors are expected to skillfully solve clinical problems early in their clinical career, they are prone to psychological pain, which has a negative impact on the formation of professional identity. In other words, because young doctors are new to clinical work, they have less medical experience than older doctors, leading young doctors to feel that their medical knowledge is poor compared with their counterparts." (lines 290–296). I hope this explanation meets your requirements.

References

1.Cohen MJM, Kay A, Youakim JM, Balaicuis JM. Identity transformation in medical students. Am J Psychoanal. 2009;69(1):43-52. doi: 10.1057/ajp.2008.38. PubMed PMID: 19295620.

2.Bynum WE, Artino AR, Uijtdehaage S, Webb AMB, Varpio L. Sentinel Emotional Events: The Nature, Triggers, and Effects of Shame Experiences in Medical Residents. Acad Med. 2019;94(1):85-93. doi: 10.1097/ACM.0000000000002479. PubMed PMID: 30277959.

Comment 8. Please proofread the grammar.

Response 8:Thank you for your constructive comment. We have proofread the grammar and made corrections as needed.

**Response to Reviewer 3**

Comment 1:This study did not mention about burnout. Please state the concept of COVID-19 burnout and burnout among physicians:

Search PubMed for: Burnout is an important public health issue at times of the COVID-19 pandemic. Current measures which focus on work-based burnout have limitations in length and/or relevance. When stepping into the post-pandemic as a new Norm Era, the burnout scale for the general population is urgently needed to fill the gap. This study aimed to develop a COVID-19 Burnout Views Scale (COVID-19 BVS) to measure burnout views of the general public in a Chinese context and examine its psychometric properties.

Search PubMed for: Our findings suggest a high prevalence of burnout among medical and surgical residents. Older and male residents suffered more than their respective counterparts.

Response 1: The journal editor stated "This manuscript does not mention burnout, so there is no need to modify the burnout mentioned by reviewer 3. Please revise other issues.” Therefore, we did not revise the text to mention burnout.

Comment 2:) The authors stated "GPs may be likely to experience mood disorders or symptoms, such as
354 depressive episodes or anxiety. If these are not addressed appropriately and in a timely
355 manner, then GPs’ psychological capital may decline". Please discuss internet psychological interventions to help GPs based on the following:

The most evidence-based treatment is cognitive behaviour therapy (CBT), especially Internet CBT that can prevent the spread of infection during the pandemic.

Use of Cognitive Behavior Therapy (CBT) to treat psychiatric symptoms during COVID-19:
Mental Health Strategies to Combat the Psychological Impact of COVID-19 Beyond Paranoia and Panic. Ann Acad Med Singapore. 2020;49(3):155‐160.

Cost-effectiveness of iCBT:
Moodle: The cost effective solution for internet cognitive behavioral therapy (I-CBT) interventions. Technol Health Care. 2017;25(1):163-165. doi: 10.3233/THC-161261. PMID: 27689560.

Internet CBT can treat psychiatric symptoms such as insomnia:
Efficacy of digital cognitive behavioural therapy for insomnia: a meta-analysis of randomised controlled trials. Sleep Med. 2020 Aug 26;75:315-325. doi: 10.1016/j.sleep.2020.08.020. Epub ahead of print. PMID: 32950013.

Response 2: Thank you for your comment. We added the following text, accordingly (lines 377–341): "Internet cognitive behavioral therapy may be an effective way to solve this problem. During the pandemic, a study in Singapore showed that an online cognitive behavioral therapy intervention could enhance people’s ability to manage stress, combat anxiety, and prevent depression, thus improving the psychological capital of GPs ."

References

1.Zhang MWB, Ho RCM. Moodle: The cost effective solution for internet cognitive behavioral therapy (I-CBT) interventions. Technol Health Care. 2017;25(1):163-5. doi: 10.3233/THC-161261. PubMed PMID: 27689560.

2. Soh HL, Ho RC, Ho CS, Tam WW. Efficacy of digital cognitive behavioural therapy for insomnia: a meta-analysis of randomised controlled trials. Sleep Med. 2020;75:315-25. doi: 10.1016/j.sleep.2020.08.020. PubMed PMID: 32950013.

3. Ho CS, Chee CY, Ho RC. Mental Health Strategies to Combat the Psychological Impact of Coronavirus Disease 2019 (COVID-19) Beyond Paranoia and Panic. Ann Acad Med Singap. 2020;49(3):155-60. PubMed PMID: 32200399.
